# Temporal and spatial analysis of enteric nervous system regeneration in the sea cucumber *Holothuria glaberrima*

**DOI:** 10.1002/reg2.15

**Published:** 2014-08-05

**Authors:** Karen Tossas, Sunny Qi‐Huang, Eugenia Cuyar, Jose E. García‐Arrarás

**Affiliations:** ^1^ Department of BiologyUniversity of Puerto RicoRio PiedrasPuerto Rico00931

**Keywords:** Digestive tract, echinoderm, neurogenesis, organogenesis, regenerations

## Abstract

There is limited information on the regeneration of the enteric nervous system (ENS) following major reconstruction of the digestive tract. We have studied ENS regeneration in the sea cucumber *Holothuria glaberrima* which undergoes an organogenic process forming a new digestive tract at the tip of the mesentery. Our results show that (1) a degeneration of nerve fibers occurs early in the regeneration process, prior to eventual regeneration; (2) nerve fibers that innervate the regenerating intestine are of extrinsic and intrinsic origin; (3) innervation by extrinsic fibers occurs in a gradient that begins in the proximal area of the regenerate; (4) late events include the appearance of nerve fibers that project from the serosa into the connective tissue and of nerve bundles in the mesothelial layer; (5) neurons and neuroendocrine cells appear early following the formation of the epithelial layers. Our results provide not only a comparative biological approach to study ENS regeneration but also an alternative point of view for the study of enteric neuropathologies and for the innervation of organs made in vitro.

## Introduction

The enteric nervous system (ENS) constitutes the ensemble of neural tissue that innervates the gastrointestinal tract. The ENS components are arranged into plexi that are located in various areas of the digestive tract. These plexi are interconnected networks made of neurons, their axons and enteric glial cells (Furness 2006). Recent findings have focused on novel roles of the ENS that extend beyond intestinal muscle contraction and peristalsis and include transmucosal fluid movement (Furness 2012) and host defense (Gulbransen & Sharkey 2012). Problems with ENS function can cause several neuropathies both neurodegenerative and inflammatory (Neunlist et al. 2012; Knowles et al. 2013). Some of these neuropathies are caused by the loss of ENS components and the resulting loss of ENS function.

Surprisingly, only a limited number of studies regarding regeneration of ENS components have been done in vertebrates, and studies in invertebrate animal models are practically absent. Stead (1992) demonstrated that, following an inflammatory response, the rat jejunal mucosal nerves undergo a degenerative and a regenerative phase that coincides with the acute and recovery stages of inflammation. Similarly, a study where incomplete surgical stenosis of the intestine was induced in dogs showed striking changes in the myenteric plexus upstream of the obstruction. The neurons innervating the muscle in the regenerating intestine increased in size and number without undergoing mitosis (Filogamo & Cracco 1995). These results were corroborated using other models where the myenteric plexus was removed chemically and the re‐innervation of the surrounding smooth muscle was studied (Hanani et al. 2003). The results suggested that the re‐innervation is due to migration of cells and that there is a reserve pool of potential neurons within the adult myenteric plexus. This is consistent with work suggesting the presence of stem cells in the rat gut (Kruger et al. 2002; Estrada‐Mondaca et al. 2007).

However, although various models have been used, these studies deal with local induced damage to the digestive tract. Noticeably absent are studies where the regeneration of the ENS is studied during regenerative organogenesis, when the complete organ is being regenerated. These studies could provide insights into two recent developments. On the one hand is the characterization of factors originating in the nervous system that are responsible for nerve‐dependent regenerative processes (Kumar & Brockes 2012). It is possible that factors similar to what have been found in other species are released from the extrinsic or intrinsic nerves and are involved in the formation of the new intestine. On the other hand is the increasing number of bioengineering studies aimed at obtaining functional intestines for transplantation (Bitar & Raghavan 2012; Orlando et al. 2012; Bitar & Zakhem 2013). Studies of ENS regeneration could provide insights into the type of cells needed or the timing at which ENS precursors should be incorporated to develop a functional organ.

Our laboratory has used a deuterostome model system, the sea cucumber *Holothuria glaberrima*, to study intestinal organogenesis. These organisms can eviscerate, and later regenerate, their internal organs (for reviews see García‐Arrarás & Greenberg 2001; Mashanov & García‐Arrarás 2011). After evisceration, mesenteries remain intact and play an important role in intestinal regeneration (García‐Arrarás et al. 1998). A primordium develops from a thickening of the mesenterial edge. As regeneration advances, the localized thickening grows in size and finally forms a continuous tube that extends from the esophagus to the cloaca. This tube becomes the regenerated intestine.

Two possible forms of nervous regeneration in the intestine have been proposed (García‐Arrarás et al. 1999). First, there is regeneration of extrinsic fibers originating in neurons outside the intestine, whose fibers enter via the mesentery. During evisceration the nerve fibers would be damaged but the somas would remain intact. New fibers emerge from the intact somas and re‐innervate the intestine. Second, new neurons appear in the regenerating structure. These new neurons give rise to intrinsic fibers within the regenerating organ.

Previous work in the laboratory characterized several components of the holothurian ENS and showed that some of them are able to regenerate (García‐Arrarás et al. 1999). However, these initial results only compared the normal uneviscerated intestine with a 21‐day post‐evisceration (dpe) regenerate, showing that all the ENS neuronal markers (available at that time) were also seen in the 21 dpe regenerated intestine. The work did not document the temporal events associated with ENS regeneration, nor was there evidence of whether fiber regeneration was extrinsic or intrinsic. Finally it remained unknown when or where neuronal cells appeared.

We have now performed a detailed analysis on the regeneration of the ENS of *H. glaberrima* using various neuronal markers. We have identified a series of events that have served to develop a spatial and temporal model of ENS regeneration. Our findings include early events of fiber degeneration that are followed by a stepwise regeneration. Both extrinsic and intrinsic fiber innervation occurs as well as the appearance of neurons and neuroendocrine cells that conform the ENS. The ENS achieves a seemingly normal cell and fiber distribution by 35 dpe. Unraveling the cellular and molecular processes involved in this degeneration and later regeneration of the ENS in the sea cucumber may help in understanding the plasticity of the ENS and its possible regeneration following loss or damage in other organisms.

## Results

### *H. glaberrima* enteric nervous system

To understand ENS regeneration in *H. glaberrima*, it is necessary to describe the organization of the normal holothurian ENS. Thus, we labeled the ENS of adult non‐regenerating animals and provide here a summary of our findings. These results confirmed previous studies from our group (Garcia‐Arraras & Viruet 1993; García‐Arrarás et al. 1999, 2001; Díaz‐Balzac et al. 2007, 2012). The main components of *H. glaberrima* ENS are divided according to their locations within (1) the mesothelial layer, (2) the connective tissue layer and (3) the luminal layer.

Before presenting the results it is important to highlight certain antibody‐labeling issues that might confound readers but that were fully addressed in a previous publication (Díaz‐Balzac et al. 2014), specifically the fact that it is not clear what antigens the antibody markers are labeling. For example, although a Pax6 homolog has been found in the *H. glaberrima* transcriptome (our own unpublished observations) the antibody against this transcription factor shows extensive labeling of neuronal fibers and somata rather than of the nuclei (where transcription factors should be found). Similarly, the PH3 antibody, which in other organisms is a marker for dividing cells, in the holothurian labels a small but specific population of nervous fibers of the enteric and central nervous system. Thus, rather than viewing the antibodies as markers for the presence of antigens corresponding to the holothurian homologs, we have used them as markers of particular cell and fiber populations to be able to follow their regeneration during the formation of a new intestine. In this way we have followed the example of many Drosophila investigators who used an antibody against horseradish peroxidase that was found to label the fly nervous system (Wang et al. 1994; Sun & Salvaterra 1995).

#### Mesothelial layer

**Figure 1:**
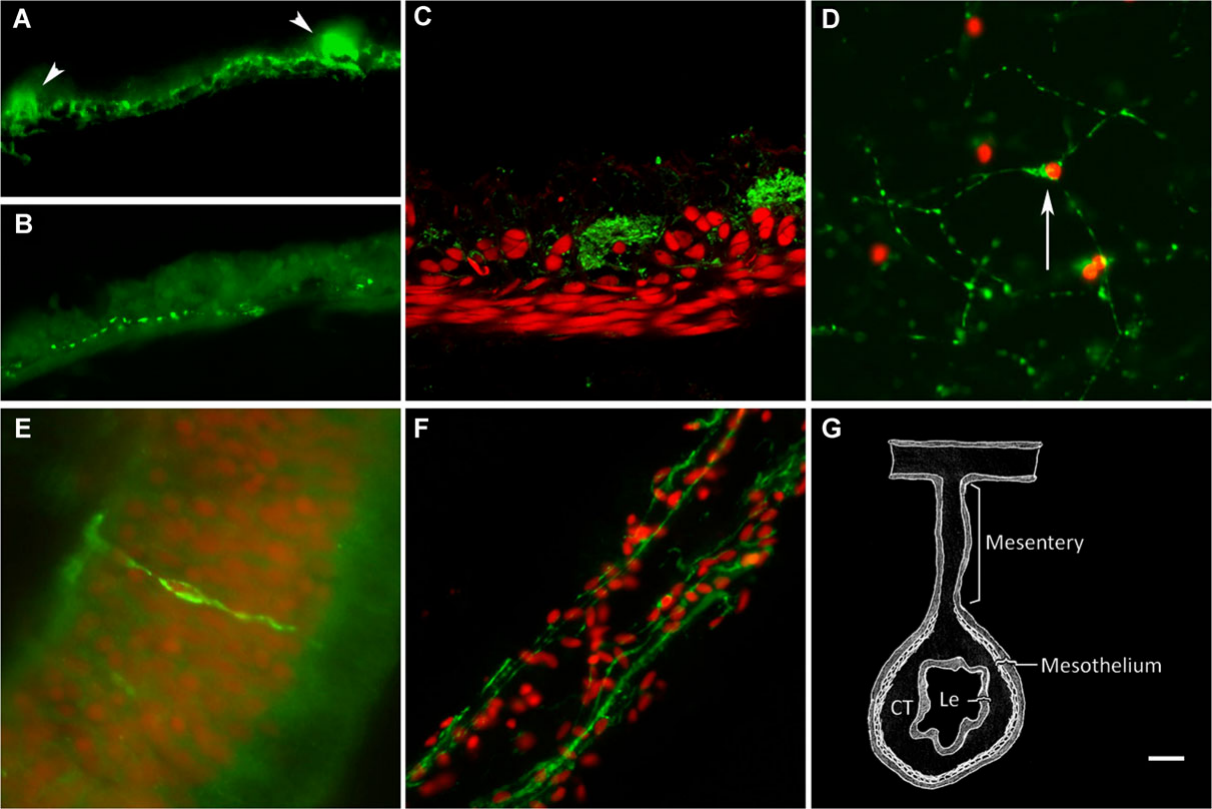
Subdivisions of the holothurian ENS. (A) Mesothelial plexus labeled with monoclonal antibody RN1 showing an extensive fiber plexus and fiber bundles (arrowhead) while (B) other markers (anti‐PH3) only labeled discrete fiber populations. (C) Confocal image showing RN1 label fiber (green) distribution in relation to the muscle layer (labeled with Cy3‐labeled phalloidin, red). (D) Connective tissue plexus as labeled by RN1. Arrow points to a neuron‐type cell. (E) Neuroendocrine cell labeled with RN1 in the luminal epithelium. (F) RN1 labeling of fibers in the mesothelial layer of the mesentery. (G) Diagram showing the intestinal tissue layers. CT, connective tissue layer; Le, luminal epithelial layer. Nuclei are stained with DAPI (red) in (D), (E), (F). Bar (A, B) 10 μm; (C, E, F) 20 μm.

The fiber plexus in the mesothelium can be divided into two main components. The first is the fiber network within the muscle layers that most probably is responsible for the innervation of the visceral muscle. These fibers, or subpopulations of them, were labeled with RN1 and antibodies against GFSKLYFamide (GFS), galanin, α‐ and β‐tubulin, acetylated α‐tubulin, calbindin and Pax6. Not all fiber subpopulations showed the same density. For example, anti‐β‐tubulin or RN1 labeled many more fibers (Fig. 1A) than those labeled by antibodies against GFS, galanin, acetylated α‐tubulin, calbindin, PH3 or Pax6, suggesting that the former antibodies may be recognizing most, or at least a large population of, enteric nerve fibers. On the other hand anti‐PH3 and anti‐Pax6 labeled very discrete fiber populations (Fig. 1B). Confocal microscope images demonstrated that the mesothelial nerve plexus was more prominently associated with the longitudinal muscle layer and provided details of individual fibers that appeared to be innervating individual muscle fibers (Fig. 1C). Neuronal cell bodies labeled with antibodies against GFS, calbindin or Pax6 were found, although in very low numbers, within the mesothelium fiber network.

The second mesothelial plexus component were large nerve bundles that run along the longitudinal axis of the intestine and that, in cross‐section, were observed as quasi‐circular structures, devoid of nuclei, and spaced regularly above the muscle layers (see Fig. 1A, C). These structures were labeled by antibodies against GFS, α‐ and β‐tubulin, calbindin, and RN1. The bundles were formed by nerve fibers that run mostly parallel to the longitudinal muscle fibers.

#### Connective tissue layer

There were two main ENS components of the connective tissue. The first were large, thick fibers perpendicular to the muscle layer (not shown) that appeared to correspond to nerves that connect the mesothelial plexus with the connective and luminal layers. These fibers were labeled by antibodies against GFS, acetylated α‐tubulin and Pax6. The second component of the connective tissue ENS was a plexus formed by a network of small neurons and fine fibers that extended throughout the connective tissue layer (Fig. 1D). This plexus was labeled by anti‐α‐tubulin and by RN1.

#### Luminal epithelium

The luminal plexus consisted of neuroendocrine‐type cells similar to those found in vertebrate intestines (Fig. 1E). These cells were distributed among the luminal epithelial cells and sometimes showed fiber‐like prolongations that entered the submucosal layer and extended for longer distances toward the mesothelium. These cell types were labeled by anti‐GFS, anti‐calbindin and anti‐Pax6.

#### Mesentery

The ENS components found within the mesentery mesothelium were basically similar to those found within the intestinal mesothelium as both tissues are continuous with each other and are made up of two main cell types, the peritoneocytes and the myocytes. Nerve fibers immunoreactive to GFS, galanin, α‐ and β‐tubulin, acetylated α‐tubulin and RN1 were found within the muscle layer (Fig. 1F). However, contrary to the intestinal tissue, no neuronal cell bodies were distinguishable in the mesentery, either in the mesothelium or within the connective tissue, nor were any large nerve bundles observed.

### The regenerating enteric nervous system

To determine the spatial and temporal pattern of ENS regeneration we labeled intestinal tissues at different stages of regeneration (3, 5, 7, 10, 14, 21, 28 and 35 dpe) with our nervous system markers.

#### ENS regeneration begins with an initial neurodegenerative event

**Figure 2:**
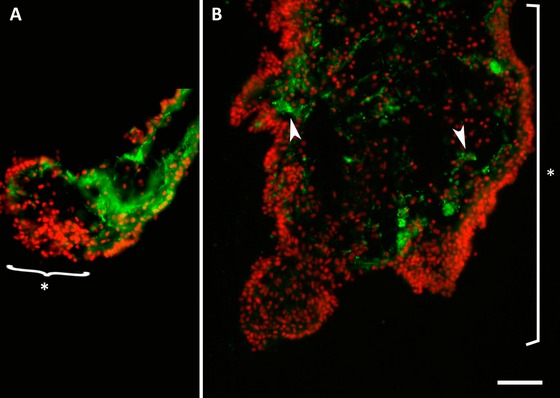
Degenerating fibers during early formation of the intestinal rudiment. (A) Fibers, labeled here with RN1 (green), are absent from the regenerating intestinal primordium at the distal tip of the mesentery (asterisk) in a 3 dpe specimen. (B) In the 5 dpe intestinal rudiment (asterisk), that has largely increased in size, there are few RN1‐labeled fibers (arrowheads) and these are disorganized and in the process of degenerating. DAPI‐stained nuclei are shown in red. Bar 50 μm.

Following evisceration, the remaining mesentery underwent dramatic changes. By 3 dpe a small thickening at the free end of the mesentery was observed (Fig. 2A). Although this region was characterized by an accumulation of cells, none of these were labeled by the nervous system markers. In the mesothelium of the remaining mesentery, nerve fibers immunoreactive to RN1 or β‐tubulin could be seen to extend from the tip of the mesentery to the attachment of the mesentery to the body wall.

As the thickening grew in size, nerve fibers identified by RN1 or anti‐acetylated α‐tubulin could be observed mostly in the center of the connective tissue layer of the regenerating portion and in the mesentery. The accumulation of nerve fibers within the connective tissue, particularly in the area of the intestinal primordium adjacent to the mesentery, became more evident at 5 and 7 dpe (Fig. 2B). However, the morphology of these fibers differed from those found within the normal intestine or mesentery. The labeling was usually beaded and interrupted rather than continuous, and the fibers showed swellings typical of degenerating fibers. In some cases, cells could be observed close to the fibers and appeared to be phagocytosing them. Moreover, the number of fibers decreased between 5 and 7 dpe and by 10 dpe no nerve fibers, or very few, were observed within the connective tissue or in the mesothelium of the intestinal rudiment.

In contrast, the neuronal fibers within the area of the mesentery that remains as a mesentery (i.e., does not widen to form the new intestinal rudiment) underwent some organization changes but were present during the overall regeneration process. Therefore in animals at about 10 dpe a transition point was observed in the region where the mesentery joins the regenerating intestinal rudiment. Neuronal fibers were present in the former as if waiting to enter the rudiment, while the rudiment itself was devoid of fibers.

#### Re‐innervation of the intestinal rudiment begins during the second week of regeneration

**Figure 3:**
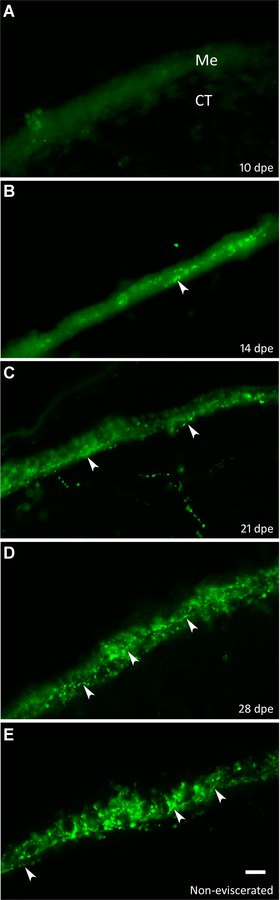
Temporal profile of the appearance of neuronal fibers in the intestinal mesothelium. Labeling with α‐GFS provides an overview of the pattern of innervation of the regenerating intestine mesothelium. (A) The mesothelium of 10 dpe is devoid of fibers. (B) Few fibers are initially observed in the mesothelium of 14 dpe specimens. The density and organization of fibers increase as regeneration progresses by 21 dpe (C). (D) At 28 dpe fiber innervation closely resembles that of (E) the normal non‐eviscerated intestine. Arrowheads signal some of the fiber varicosities in the mesothelium. Me, mesothelial layer; CT, connective tissue layer. Bar 10 μm.

Early during the second week of regeneration the intestinal rudiment mesothelium was devoid of fibers (Fig. 3A). By 14 dpe the beginning of re‐innervation of the intestinal rudiment was observed (Fig. 3B). Immunoreactive nerve fibers were seen with all markers (RN1, and anti‐PH3, anti‐β‐tubulin, anti‐GFS, anti‐calbindin, anti‐galanin, and anti‐Pax6). Fibers were few and isolated and were mainly found in the basal area of the mesothelium where the muscle layer was beginning to form. Two different patterns of nerve fiber regeneration were observed. The first was observed using anti‐GFS and anti‐galanin, where nerve fibers were found throughout the mesothelium of the regenerating intestine distributed homogeneously along proximal or distal areas. The density of fibers appeared to increase from 14 to 28 dpe approaching a density similar to that in the normal, non‐eviscerated intestine by the latter date (Fig. 3C−E).

**Figure 4:**
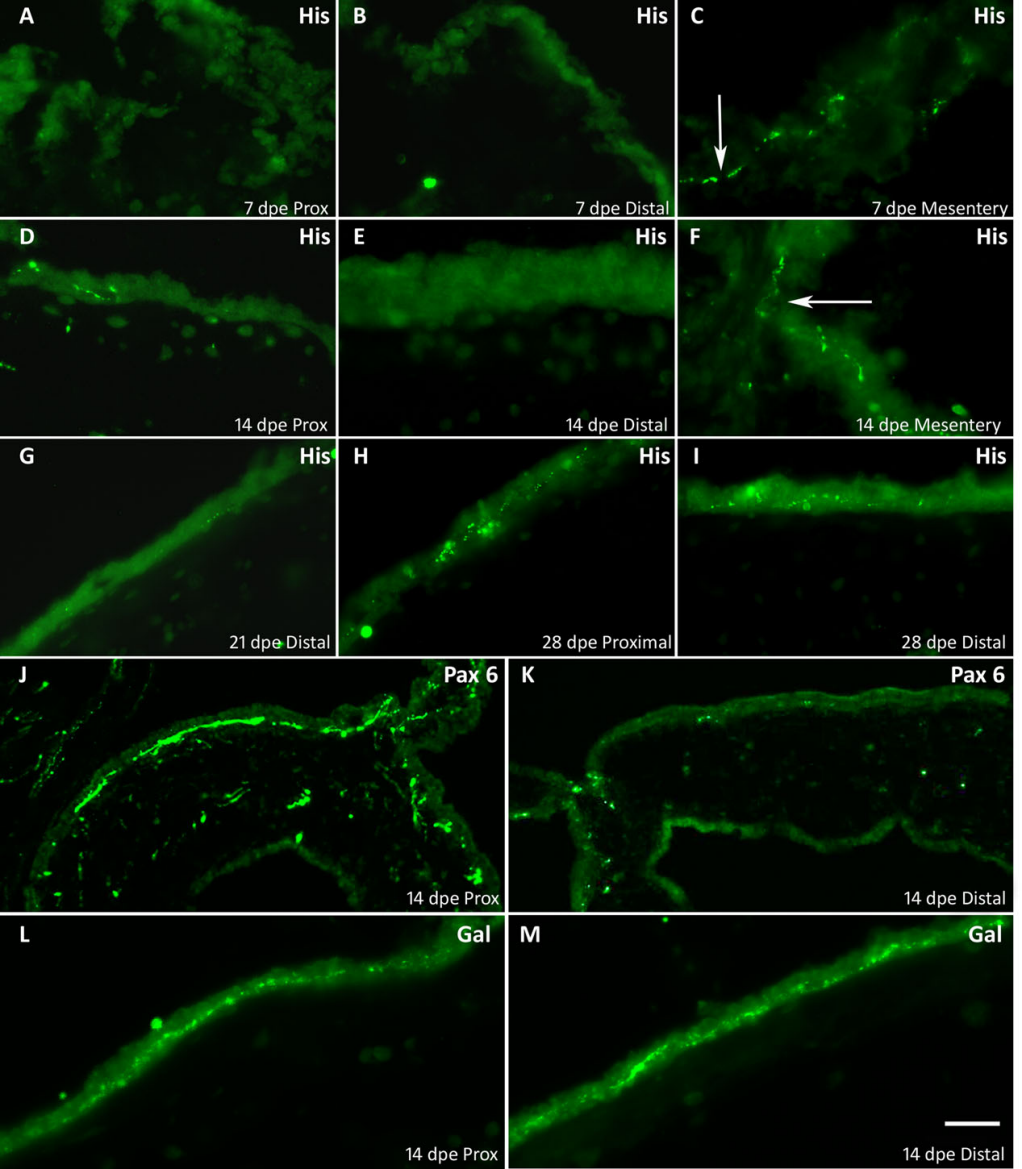
Differential innervation pattern between the distal and proximal areas of the regenerating rudiment. In 7 dpe regenerating intestinal rudiment nerve fibers immunoreactive to α‐PH3 (His) are not present in (A) proximal or (B) distal mesothelium, but can be found in (C) the adjacent mesentery. At 14 dpe, PH3‐immunoreactive fibers are found in (D) the proximal but not in (E) the distal mesothelium of the intestinal rudiment. (F) PH3‐immunoreactive fibers are particularly abundant in the connecting region of the mesentery and the intestinal rudiment. A few PH3‐immunoreactive fibers are observed in (G) the distal 21 dpe rudiment mesothelium (arrow) and by 28 dpe fibers are found in both (H) proximal and (I) distal mesothelium. Differential innervation by Pax6‐immunoreactive fibers in (J) the proximal mesothelium of 14 dpe intestinal rudiments but not in (K) the distal portion. Notice the abundant fiber immunoreactivity (arrow) at the mesentery intestinal junction. Anti‐galanin‐immunoreactive fibers can be found in the mesothelium of both (L) proximal and (M) distal mesothelium of 14 dpe intestinal rudiments. Bar 50 μm.

The second innervation pattern was shown by anti‐Pax6‐ and anti‐PH3‐labeled fibers. These immunolabeled fibers showed a small lag in their distribution pattern between the proximal and distal areas of the intestinal rudiment. Using immunoreactivity to PH3 as an example, we observed that early in regeneration (7 dpe) no fibers were found in either proximal (Fig. 4A) or distal (Fig. 4B) areas of the rudiment mesothelium, while they were present in the mesothelium of the adjacent mesentery (Fig. 4C). As regeneration continued (14 dpe), a few fibers were first observed in the mesothelium of the proximal area of the intestinal rudiment (Fig. 4D) but not in the distal area (Fig. 4E). Immunoreactive fibers could be seen connecting the mesothelial plexus of the mesentery with those of the proximal area of the rudiment (Fig. 4F). Fibers were first observed in the mesothelium of the distal area of the rudiment during the third week of regeneration (Fig. 4G) and by the fourth week of regeneration both proximal (Fig. 4H) and distal (Fig. 4I) areas were innervated. The initial distribution suggested that these fibers were of extrinsic origin and that they entered into the regenerating structure via the mesentery and eventually grew toward the distal part of the rudiment.

The difference in innervation patterns was also evident when comparisons were made between fibers immunoreactive to anti‐Pax6 and those immunoreactive to anti‐galanin. During the second week of regeneration, the former were found almost exclusively in the proximal area of the growing intestinal rudiment (Fig. 4J) with little or no immunoreactivity in the distal area (Fig. 4K). In contrast, anti‐galanin‐immunoreactive fibers were found in both proximal (Fig. 4L) and distal (Fig. 4M) areas.

**Figure 5:**
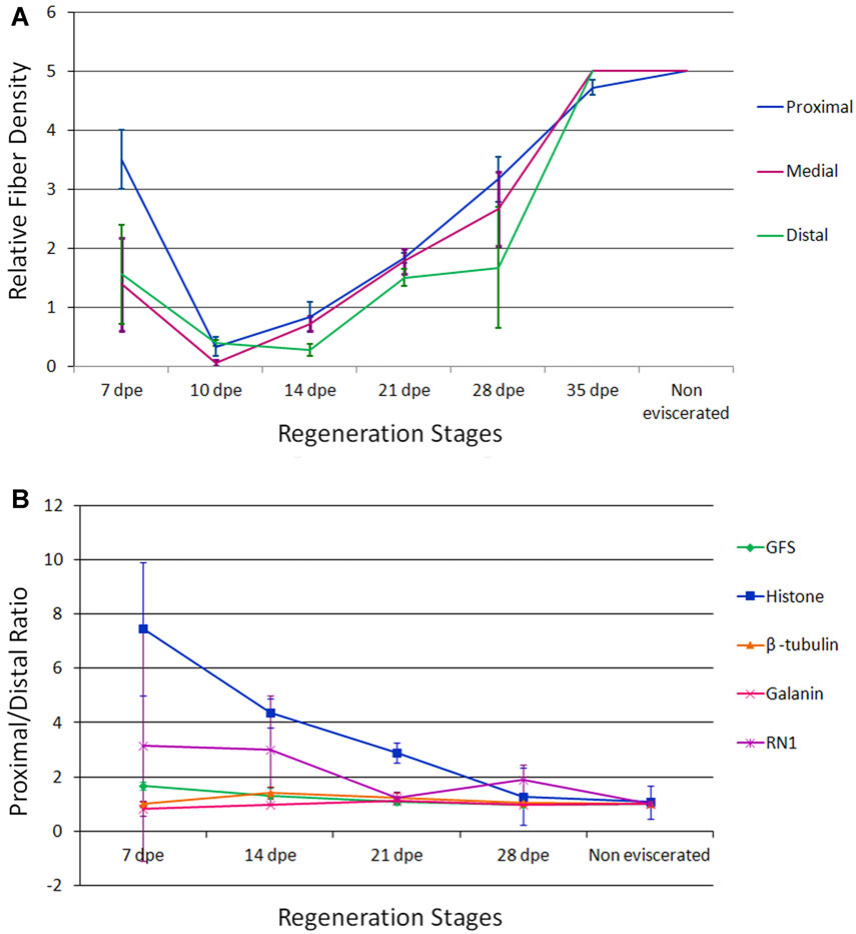
Quantification of fiber regeneration in different regions of the intestinal rudiment. (A) Relative density of RN1‐labeled nerve fibers in the mesothelium of the intestinal rudiment during the various stages of regeneration and in the non‐eviscerated (normal) specimens. The relative amount of fibers practically disappears during the early stages and then regenerates to levels similar to normal by 35 dpe. Each point represents the mean ± SD of at least three animals. (B) Proximal/distal fiber density ratios for various fiber populations. Fibers were labeled with antibodies against GFS, galanin, β‐tubulin, PH3 (histone) or with monoclonal antibody RN1. See Materials and methods for quantification procedure. Each point represents the mean ± SE of at least three animals.

Nerve fiber appearance in the mesothelium was quantified using anti‐PH3, anti‐β‐tubulin, anti‐GFS, anti‐galanin, and RN1 at various stages of regeneration. In general terms a similar pattern of regeneration was observed where the innervated area decreased from 7 to 10 dpe and then increased slowly as regeneration advanced (Fig. 5A). For some of the labeled fibers a difference was observed in the labeling of proximal areas compared with distal areas. To quantify this difference in innervation patterns we determined the distal versus proximal ratio of fiber innervation (Fig. 5B). The proximal/distal ratios (close to 1) obtained using antibodies against β‐tubulin, GFS, and galanin were maintained throughout regeneration, suggesting that these fibers are homogeneously distributed in the mesothelium of the forming intestinal rudiment. In contrast, anti‐His demonstrated a higher proximal/distal ratio during early stages of regeneration and a decrease as regeneration advanced, reaching values close to 1 by 28 dpe. A pattern between these two was shown by RN1 with a less marked difference than anti‐PH3 in the earlier stages of regeneration that eventually disappeared by the third week of regeneration.

#### Regeneration of the connective tissue plexus

The appearance of nerve fibers in the connective tissue during regeneration was also observed using anti‐RN1, anti‐Pax6, and anti‐acetylated α‐tubulin. These fibers also showed a spatial regeneration gradient where in the 14 dpe animal fibers were observed in the proximal areas but not in the distal areas (not shown). Quantification of the fiber distribution showed that the proximal/distal ratio was high (9) at 14 dpe and then decreased, reaching values of 1 by 21 dpe (RN1 and acetylated α‐tubulin) and 28 dpe (Pax6).

**Figure 6:**
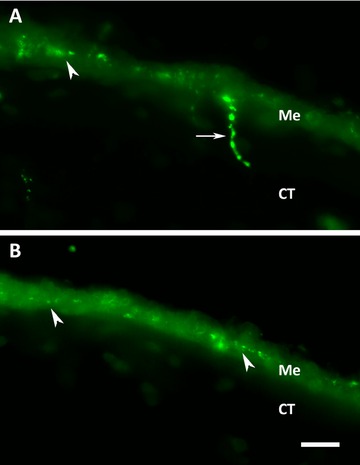
Regeneration of perpendicular fibers in the connective tissue of the intestinal rudiment. These varicose fibers (arrow) that cross from the mesothelium into the connective tissue are first observed in the proximal area of the rudiment by 14 dpe (A) using the RN1 antibody, while they are absent in the distal area of the rudiment at the same stage (B). Arrowheads signal some of the fiber varicosities in the mesothelium. Me, mesothelial layer; CT, connective tissue layer. Bar 20 μm.

We also studied a second connective tissue ENS component made up by nerve fibers that project perpendicular from the mesothelium into the underlying connective tissue. These fibers were first observed at 14 dpe in the proximal (Fig. 6A) but not in the distal (Fig. 6B) areas of the intestinal rudiment. The presence of these fibers was quantified using anti‐acetylated α‐tubulin. The results showed that this component appeared between 14 and 21 dpe for proximal and medial portions of the regenerate and between 21 and 28 dpe for distal portions.

#### Regeneration of nerve bundles is a late event

**Figure 7:**
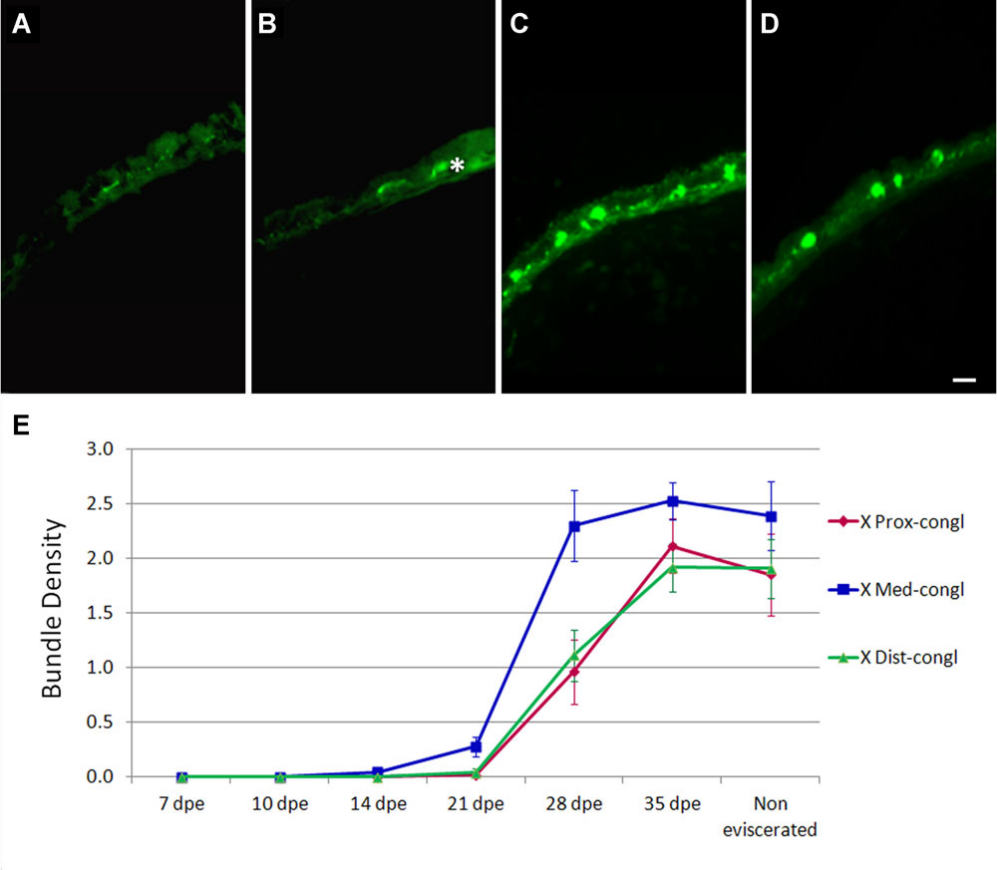
Regeneration of fiber bundles. Anti‐β‐tubulin immunoreactivity shows the late appearance of fiber bundles during intestinal regeneration. The bundles are not present in (A) 14 dpe intestines and can be seen to begin to organize in (B) 21 dpe intestinal regenerates. The asterisk (*) shows nerve fibers at different focus planes that might appear to be nerve bundles. (C) At 28 dpe the bundles are organized similarly to those in (D) the normal uneviscerated intestines. (E) Quantification of bundles using immunoreactivity to β‐tubulin shows a sharp rise between 21 and 28 dpe that appears to take place faster in the medial section of the rudiment. Bar 10 μm. Each point represents the mean ± SD of at least three animals.

Clearly organized nerve bundles were not observed in 14 dpe animals (Fig. 7A). The first indication of bundle formation was observed in 21 dpe regenerating intestines (Fig. 7B), and by 28 dpe bundles were clearly observed (Fig. 7C) and were similar in morphology and density to those observed in normal (non‐eviscerated) animals (Fig. 7D). The nerve bundles above the muscle layer first appeared in the medial portion of the regenerate by 21 dpe and by 28 dpe in the proximal and distal portions. Their presence was quantified using anti‐β‐tubulin and RN1. With both markers, the number of fiber bundles presented a steady rise from 21 to 35 dpe (Fig. 7E). At this last stage, the values in all three portions of the regenerate were similar to those of the normal non‐eviscerated intestine.

#### The mesentery mesothelial plexus shows little change

Nerve fibers in the mesothelial layer of the mesentery were quantified using antibodies RN1 and anti‐GFS, anti‐galanin, and anti‐PH3. While there were some minor changes in the general organization and morphology of nerve fibers within the mesentery the fiber distribution and abundance did not appear to change markedly.

#### Cells with neuronal properties appear within the regenerating intestine

**Figure 8:**
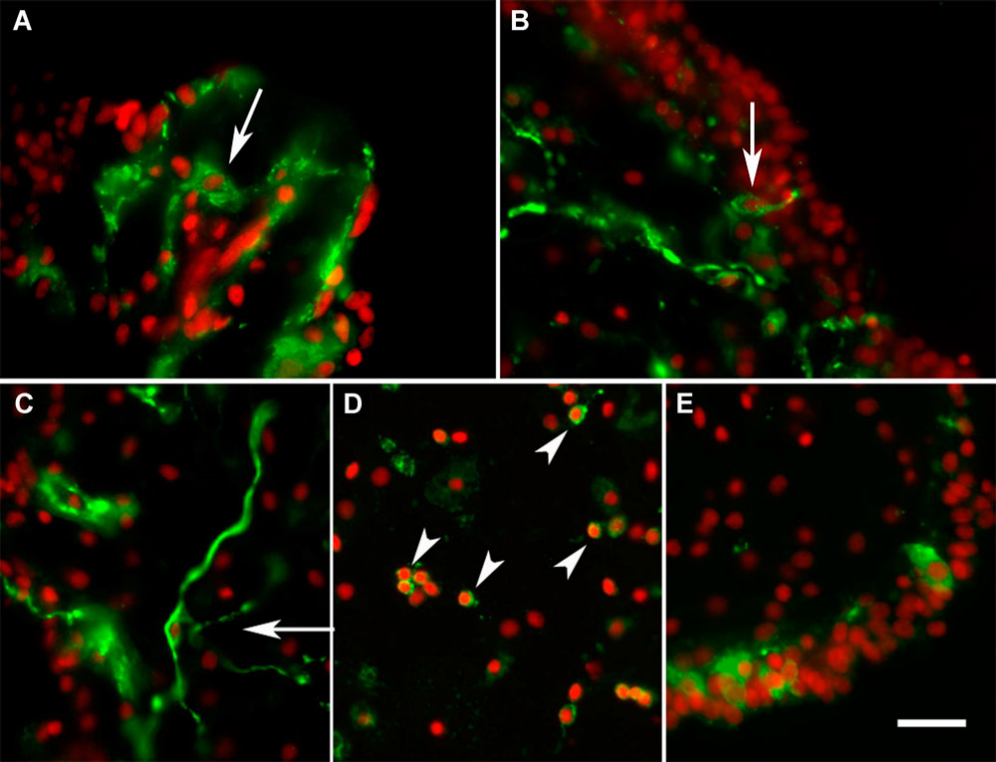
Regeneration of cells labeled with neuronal markers. Cells labeled with neuronal markers are not observed in the normal mesentery; some cells labeled with RN1 (green) appear during the early stages of regeneration in the mesentery mesothelium at (A) 3 and (B) 5 dpe (arrows). (C), (D) RN1‐labeled cells are also found within the connective tissue of the growing rudiment and (C) some show long neurite‐like extensions (arrow) while others (D) are small round cells (arrowheads) weakly labeled by the antibody. (E) Immunoreactivity to calbindin (green) shows the initial appearance of cells in the mesothelium of a 10 dpe specimen. DAPI nuclei stain (red). Bar 20 μm.

The first cells labeled with neuronal markers were observed in animals at 3 and 5 dpe using RN1 and β‐tubulin labeling. These cells were found in the mesentery adjacent to the thickening and, in particular, in the area of the mesothelium close to the connective tissue or within the connective tissue itself (Fig. 8A, B). This labeling was intriguing since these cells are not observed in normal (uneviscerated animals). The appearance of the cells was transitory; by 7 dpe the number of cells in the mesothelium had decreased and they were not observed at 14 dpe or later stages. Other RN1‐immunoreactive neuron‐like cells, that clearly showed a neuronal morphology, were found within the connective tissue of the mesentery at 7 and 14 dpe (Fig. 8C). These cells had a small soma and long fiber extensions that were oriented along the mesentery proximal−distal axis. They were not found in the mesentery at more advanced regeneration stages nor in normal animals, but were eventually found within the connective tissue of the intestinal rudiment, first in the proximal area close to the mesentery and then in both proximal and distal areas of the rudiment. A second RN1‐immunoreactive cell type was found in the connective tissue of the rudiment. These cells were small with little cytoplasm and showed no or few neurites (Fig. 8D). They were present from 10 dpe (Table 1), their numbers increasing up to 28 dpe and then decreasing at 35 dpe.

**Table 1:** Presence of neuron populations in individual animals at different regenerative stages

	Calbindin		RN1		PAX 6		GFS	
		Connective		Connective		Connective		Connective
Stages	Mesothelium	tissue	Mesothelium	tissue	Mesothelium	tissue	Mesothelium	tissue
10 dpe	+ + +	+ − −	+ *	+ *	− − −	− − −	+ − −	+ − −
14 dpe	+ + +	+ − −	+ + +	+ + +	− − +	− − −	− − −	− − +
21 dpe	+ + +	+ − −	+ + −	+ + +	− − +	− − −	+ − +	+ − −
28 dpe	+ + +	− + −	+ − −	+ + +	− + +	− − −	+ + +	− − −
35 dpe	+ + +	− − −	− − −	+ + +	+ + −	− − −	+ + −	− − −
Normal	+ + +	− − −	− − −	+ + +	+ + +	− − −	+ + +	+ − −

+, neurons were present in the tissue slides of one organism.

−, neurons were absent in the tissue slides of one organism.

*Only one organism was observed at this stage.

Cells with neuronal properties were also found in the mesothelium of the regenerating intestine. However, cells in this tissue layer can be few and hard to find which causes some variability in our results (Table 1). Nonetheless, the first neuron‐like cells in the mesothelium of the regenerating intestine were observed between 10 and 14 dpe labeled by RN1 or anti‐calbindin (Fig. 8E). The appearance of GFS‐ and Pax6‐labeled cells occurred at more advanced stages of regeneration (21 and 28 dpe) and coincided with the disappearance of the RN1‐labeled cells, suggesting that the latter cell type was giving rise to the others. By 35 dpe the expression profile of neuronal markers in the mesothelial layers was strikingly similar to that found in normal intestines (Table 1). We could not distinguish any spatial pattern in the appearance of the mesothelial neuron‐like cells as they seemed to be homogeneously distributed in different areas of the regenerate, including proximal, medial, and distal regions, without any preferential localization at a particular regenerative stage.

#### Neuroendocrine cells appear soon after lumen formation

**Figure 9:**
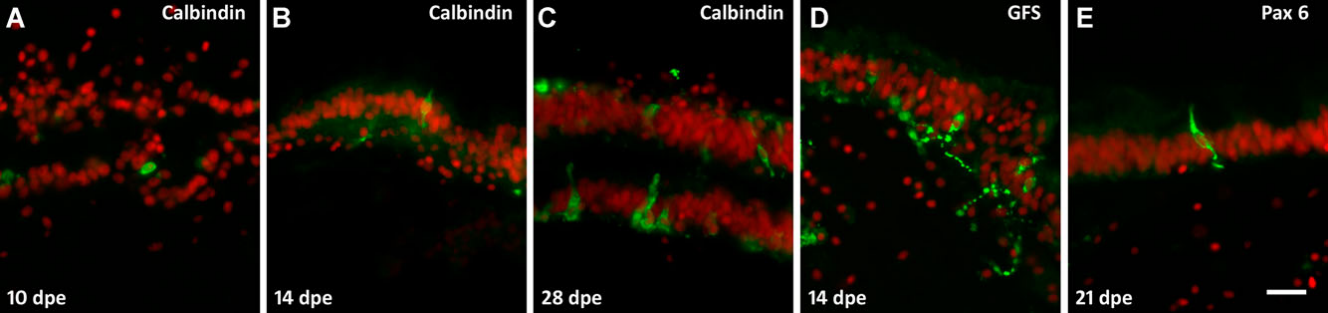
Regeneration of neuroendocrine cells. (A) Initial appearance of calbindin‐immunoreactive cells in a 10 dpe luminal epithelium is observed soon after lumen formation. At 14 dpe (B) calbindin‐immunoreactive cells acquire neuroendocrine morphology and by 28 dpe (C) the number of cells has increased significantly (the figure shows two adjacent luminal epithelia). GFS‐immunoreactive cells (D) also appear during the second week of regeneration showing their extended fiber connections. (E) Pax6‐immunoreactive neuroendocrine cells appear at 21 dpe. Bar 20 μm.

Neuroendocrine cells appeared very early, concomitant with the formation of the luminal epithelium, and were observed in the intestine of 10 dpe regenerates, the first specimens that showed luminal epithelia. However, not all neuroendocrine cells appeared simultaneously. At 10 dpe, only anti‐calbindin‐immunopositive cells were observed in the luminal layer (Fig. 9A). Interestingly, these calbindin‐immunoreactive cells showed a circular morphology and lacked fiber extensions, suggesting that they were still undergoing differentiation from luminal precursors. In contrast, at later stages, calbindin‐labeled cells showed the characteristic neuroendocrine morphology (Fig. 9B, C). Cells labeled with anti‐GFS with their oval shape and fiber extensions through the epithelium were found at 14 dpe (Fig. 9D) and anti‐Pax6‐immunoreactive cells were first identified at 21 dpe (Fig. 9E).

## Discussion

Our findings corroborate previously published data and add qualitative and quantitative data on the temporal and spatial events involved in ENS regeneration. The following discussion highlights some of the findings and their relevance to the study of ENS and overall regeneration of the nervous system.

### Regeneration of the enteric nervous system

#### Neurodegeneration precedes neuroregeneration

Our experimental results demonstrate that as the mesentery thickening grows in size to give rise to the intestinal rudiment, a neurodegenerative process takes place where neuronal elements are actively degraded. Thus, by 10 dpe, nerve fibers within the mesothelium degenerate almost completely as evidenced by an absence of immunoreactivity to any neuronal markers within the regenerating intestinal rudiment. This degeneration phenomenon has been described in other animal models of intestinal regeneration after induced lesions or evoked by inflammation in cases of inflammatory diseases. In the work by Hanani & colleagues (2003) the myenteric plexus of the mouse colon was injured with detergent. By the second day after treatment, they observed a neurodegeneration in the transition zone adjacent to the treated area that preceded the regenerative process. Similarly, following a crush of the postganglionic sympathetic nerves in the mouse digestive system, nerve fibers and neuronal bodies of both enteric plexi exhibited a degenerative response prior to regeneration. This was evidenced by a complete disappearance of tyrosine hydroxylase immunoreactivity starting as early as day 1 after nerve crush (Yamada et al. 2006). Finally, a degenerative phase was also observed in the ENS response to intestinal inflammation, in which damage to peripheral nerves caused degeneration and initiated axonal regeneration and sprouting, resulting in successful re‐innervation (Stead 1992). In patients with chronic inflammatory diseases, such as Crohn's disease and ulcerative colitis, the ENS structural changes are often accompanied by ganglion cell and nerve process degeneration and necrosis, showing swollen, empty axons filled with large membrane‐bound vacuoles, swollen mitochondria and concentrated neurofibrils (Vasina et al. 2006). Similar findings have been shown with acute intestinal inflammation where, immediately following initial stages of acute inflammation, intestinal nerves degenerate, eventually regenerating days after this initial response (Belkind‐Gerson et al. 2006). Therefore, the degenerative−regenerative process documented in the sea cucumber seems to be homologous to the degeneration−regeneration that follows induced lesions or inflammatory responses in vertebrates.

It is also noteworthy that a clearly visible transition zone separates the mesentery from the regenerating rudiment. Fibers are found in the former but degenerate in the latter. Such striking differences suggest that the initial steps in organ regeneration might be under the influence of adjacent nerve fibers as has been shown for other regeneration processes (Kumar & Brockes 2012). Nonetheless, it also suggests that certain events in intestinal regeneration must take place prior to the innervation of tissues by extrinsic fiber components.

#### Extrinsic versus intrinsic innervation

Our results demonstrate the process by which the regenerating intestine is innervated. This process begins during the second week of regeneration and continues uninterrupted until the fiber and cell distribution that makes up the regenerated intestinal ENS closely parallels that found in the normal, non‐eviscerated animals. This regenerative response is therefore similar to that observed in the vertebrate system following injury. For example, a week after the degenerative event, growth cones and elongated glial cells were found in the mouse myenteric plexus (Hanani et al. 2003). These fibers continued growing and extending throughout the injury site and by day 60 nerve fibers had re‐innervated the muscle. Similarly, a week following a crush of the extrinsic innervation, growing fibers were observed within the intestine, and eventual re‐innervation and recovery of catecholaminergic immunoreactivity was observed by 90 days (Yamada et al. 2006).

The results of the nerve fiber quantification experiments in which a ratio was established between proximal and distal portions suggest that the regenerative event encompasses two different populations of fibers. The fact that the ratios of fibers labeled with anti‐PH3 are greater than 1 during early stages of regeneration and become close to 1 as regeneration advances suggests that these are extrinsic fibers, arriving via the mesentery, entering the proximal portion of the regenerate and extending towards the distal portions as regeneration advances. We have recently shown that neuronal somas labeled for Pax6 or anti‐PH3 are found outside the digestive tract, in the peripheral and radial nerves (Díaz‐Balzac et al. 2014), providing further support to the hypothesis that these nerve fibers are of extrinsic origin. Immunohistochemical evidence demonstrates that this is a very specific population of fibers found in the mesentery that runs between the two muscle layers of the intestine and connects to the intrinsic nerve bundles in the serosa. This is homologous to what is observed in the mammalian ENS, in which extrinsic nerve fibers from the paravascular and perivascular nerves enter the intestine and connect with the different plexi or to the intramural pelvic nerves that run between the longitudinal and circular muscle layers of the large intestine (Furness 2006; Brookes et al. 2013).

Quantification results also suggest that extrinsic nerve fibers are entering the connective tissue via the mesentery; this entrance occurs mostly by 14 dpe. Extrinsic fibers are also possibly responsible for much of the growth of the nerve bundles and the nerve fibers that project from the mesothelium into the connective tissue. These will probably establish connections between the mesothelium and other ENS plexi of the sea cucumber.

#### ENS regeneration occurs concomitant with regeneration of other cell and tissue layers

Our results also strengthen the notion that ENS regeneration occurs in parallel with regeneration of other intestinal tissues. There are several events that although at present can only be coincidentally associated might involve a causative relationship that should be studied in future studies.

First in the regeneration timeline is the neurodegeneration of the neuronal fibers. This event coincides with the observed remodeling of the ECM (Quiñones et al. 2002). Therefore the formation of the intestinal rudiment apparently requires a “cleaning of the slate” and removing cellular as well as extracellular components in order to be able to form the new components. In this respect, phagocytosing cells that have been previously observed to eliminate collagen are also probably eliminating the neuronal fiber debris. It is also interesting that an increase in apoptosis in the mesothelium has been documented during this period (Mashanov et al. 2010). This coincides with the time at which no nerve cell bodies were found in the regenerating structure, suggesting that not only nerve fibers but also neuronal somas are being actively eliminated prior to the regenerative event.

Fiber regeneration coincides with the process of myogenesis, when new muscle cells are formed from precursor cells possibly found in the coelomic epithelia (Murray & García‐Arrarás 2004; Candelaria et al. 2006). The incoming fibers are possibly involved in the re‐innervation of the forming muscle layer. The increase in muscle layer thickness comes hand in hand with an increase in fiber density, although it seems that muscle formation and growth is occurring slightly ahead of the nervous fiber innervation. Similarly, the appearance of neurons within the mesothelium appears to be a bit delayed in comparison to myogenesis.

The late events in ENS regeneration also coincide with the formation or organization of other tissue layers. The appearance of the nerve fibers oriented perpendicular to the mesothelium coincides with the formation of the lumen, while the formation of the nerve bundles takes place when the muscle has organized into longitudinal and circular layers.

#### Possible diverse origins for ENS cells

Our results show that, not only fibers, but also cells of the ENS are regenerating during the formation of the new intestines. Moreover, the appearance of these cells occurs concomitant to the formation of the intestinal tissue layer and the appearance of neuronal fibers. However, our results suggest that neuronal cells of the regenerated intestine might have different origins. For instance, mesothelial plexus neurons could be originating from still undefined neuronal stem cells in the mesentery or, as an alternative, from cells that have been shown to dedifferentiate in the mesenterial mesothelium (García‐Arrarás et al. 2011). It is known that in holothurian not only are muscle cells able to dedifferentiate but other cell types such as epithelial and glia are also able to lose some of their cellular components and transform into a seemingly more “plastic” phenotype (Mashanov et al. 2008). Therefore, it is tempting to speculate that dedifferentiating muscle cells or coelomic epithelial cells are giving rise to the mesothelial neurons. In fact, such differentiation of coelomic epithelia into neurons of the mesothelium has been proposed before in other echinoderm model systems, although there is no solid evidence to support this (VandenSpiegel et al. 2000). An alternative origin is the possibility that the intestinal tissue or mesentery has enteric stem cells or glia that might give rise to the new neurons. This alternative is supported by findings from our group showing that in the radial nerve cord glial cells appear to be the precursor of neurons following injury (Mashanov et al. 2013). However, the lack of markers needed to identify these cells in the echinoderm tissues prevents us from analyzing this possibility. In mammals, both glia and ENS stem cells have been shown to be able to give rise to neurons of the digestive tract (Schäfer et al. 2009; Laranjeira et al. 2011; Wood 2011).

Several findings suggested the possibility that ENS precursors in the mesentery migrated into the intestinal rudiment and gave rise to the neuronal population within the connective tissue of the new intestine. First, the cellular morphology that extends along the mesentery−rudiment axis is characteristic of migrating cells. Second, the appearance of neuron‐like cells in the rudiment's connective tissue followed the proximal−distal profile observed for fibers. Third, the appearance of the “migrating” cells coincided with the later appearance of small RN1‐labeled cells in the connective tissue that showed few or no neurite‐like extensions suggesting that once within the intestinal rudiment the “migrating” cells gave rise to other cell types. Nonetheless, the possibility still exists that the cells in the mesentery connective tissue themselves originate from the dedifferentiating cells in the mesothelium. This will explain the presence of neuronal‐labeled cells in the mesothelium of 2 and 5 dpe animals that eventually disappear as regeneration advances.

In contrast, the origin of the neuroendocrine cells is possibly from the luminal epithelial cells themselves. These cells are first observed soon after luminal cell layer formation. This pattern suggests that some luminal cells are differentiating into neuroendocrine cells very early in the regenerative process. Such an event would be similar to what takes place in the mammalian luminal epithelium where a precursor found in the intestinal crypts gives rise to all the different types of luminal cells, including endocrine cells (Cheng & Leblond 1974; Simons & Clevers 2011).

#### ENS regeneration mode

**Figure 10:**
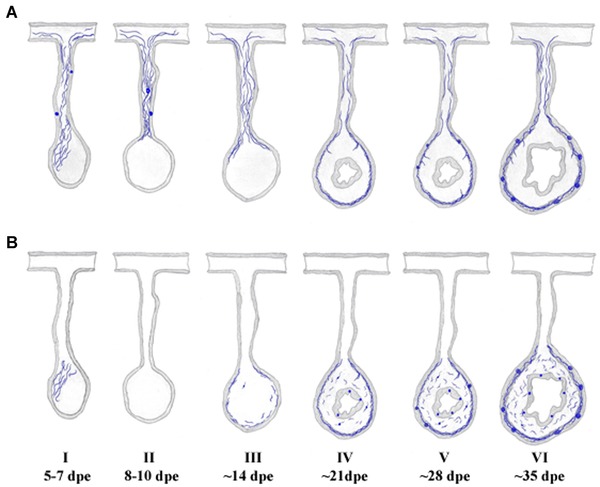
Temporal stages of enteric nervous system regeneration. (A) Extrinsic innervation occurs via the mesentery. Nervous fibers remain within the mesentery even when most of the nervous innervation to the forming intestinal rudiment is degraded and lost. As regeneration continues, fibers originating in neurons present in the mesentery or other tissues such as the radial nerve (not shown) re‐enter the proximal regions of the new intestinal rudiment and eventually arrive at the distal regions covering the complete intestinal rudiment. The extrinsic innervation preferentially involves the muscle in the mesothelial layers. (B) Intrinsic innervation. Neuron‐like cells appear during the second week of regeneration in the mesothelium, connective tissue and luminal epithelial layers. Fibers originating from these neurons are also found within these tissues, their density increasing as the new intestine forms and acquires the characteristics of the normal mature intestine. Formation of nervous bundles is shown in both extrinsic and intrinsic innervation panels, since their formation most probably involves both components.

Our experimental results provide a working model of the spatial and temporal events that take place during intestinal regeneration and in particular those that involve the ENS (Fig. 10). The initial event is a local neurodegeneration where the fiber network present at the distal tip of the mesentery disappears as the area thickens to form the regenerating intestinal rudiment (Fig. 10A‐I, B‐I). The outcome of this degenerative event is that few or no fibers can be observed in the rudiment prior to its re‐innervation (Fig. 10A‐II, B‐II). Two types of re‐innervation processes were observed, and both appear to take place concomitantly. First, extrinsic fibers originating from cells in the mesentery, body wall, or radial nerve extend their fibers through the mesentery and enter the proximal area of the intestinal regenerate (Fig. 10A‐III). This event takes place during the second week of regeneration and proceeds during the next weeks as the fibers grow into the distal areas of the regenerate (Fig. 10A‐IV). The second event is the regeneration that takes place by intrinsic neurons of the regenerating intestine. In this case fibers appear as new neurons themselves are differentiating and they appear in both proximal and distal areas of the regenerate (Fig. 10B‐III−B‐V).

More importantly, our results provide an outline of the steps or stages that take place during ENS regeneration of the holothurian intestine. The first stage (Fig. 10, I, 5−7 dpe) is a neurodegenerative stage where the fiber network found within the expanding tip of the mesentery is degraded. This stage evolves into a second stage (Fig. 10, II, 8−10 dpe) where the intestinal primordium lacks any type of ENS innervation. This denervated stage represents a short, transitory stage soon followed by the initial re‐innervation of the regenerate. This third stage (Fig. 10, III, ∼14 dpe) is characterized by the appearance of nerve fibers in the mesothelium from both extrinsic and intrinsic sources, where the former are preferentially found in the proximal areas of the regenerate. Cells and cell fibers are also found in the connective tissue, also primarily in the proximal areas. The appearance of large fibers that cross from the mesothelium into the connective tissue characterizes the fourth stage of regeneration (Fig. 10, IV, ∼21 dpe) where the mesothelial layer remains partially innervated and some differences in innervation between the proximal and distal areas can still be found. By the fifth stage (Fig. 10, V, ∼28 dpe) most of the mesothelium is innervated and differences between the proximal and distal areas have disappeared. Large fibers crossing from the mesothelium into the connective tissue can be found throughout the intestinal regenerate and the first large fiber bundles are found in the mesothelium particularly in the midsection of the intestinal regenerate. The sixth and last stage (Fig. 10, VI, ∼35 dpe) is mainly characterized by an ENS that is similar to that found in normal non‐eviscerated intestines and particularly by the presence of fiber bundles throughout the circumference of the new intestine.

## Materials and methods

### Animals

Adult specimens of *H. glaberrima* between 10 and 15 cm were collected from the northeastern coast of Puerto Rico. All experiments were performed in accordance with federal and university guidelines and regulations. Experimental animals were eviscerated by injecting 3−4 mL (0.35 mol/L) KCl intracoelomically. Regenerating and control (non‐eviscerated) animals were maintained in a seawater aquarium at 20−24°C under controlled salinity. Eviscerated animals were allowed to regenerate for 3, 5, 7, 10, 14, 21, 28 or 35 days according to the experiment.

### Immunocytochemistry

Immunocytochemical techniques were performed according to García‐Arrarás (1993). In brief, animals were dissected and intestines were fixed overnight in 4% paraformaldehyde at 4°C, washed three times in 0.1 mol/L phosphate buffered saline (PBS) at pH 7.4 and left overnight at 4°C in a 30% sucrose/0.1 mol/L PBS solution. Tissues were embedded in Tissue Tec and 15−30 μm tissue sections were cut in a Leica cryostat, mounted on slides coated with polylysine and dried under cold air. For polyclonal antibodies, slides were treated with anti‐goat serum for 1 h at room temperature, washed once in Triton/0.1 mol/L PBS for 15 min and then washed twice in PBS 0.1 mol/L before antibody treatment. Primary antibodies were left overnight in a humid chamber at room temperature. Slides were then washed twice with 0.1 mol/L PBS, treated with secondary antibody for 1 h and washed twice with 0.1 mol/L PBS. In some cases slides were also treated with Hoechst nuclear dye for 10 min and then washed twice in 0.1 mol/L PBS for 15 min. For double labeling experiments, antibodies were mixed in equal volumes to obtain the desired final concentrations. Slides were mounted with coverslips using buffered glycerol and examined under a Leitz Laborlux microscope, a Nikon Eclipse E600 fluorescent microscope and/or a Zeiss LSM 510 confocal microscope equipped with a MIRA 900 IR laser powered by a Verdi V10 pump Coherent.

Various primary monoclonal antibodies were used. Commercial antibody α‐β‐tubulin clone TUB 2.1 (Sigma T4026, St. Louis, MO) prepared from purified rat brain tubulin was used at a 1:500 dilution (Gozes & Barnstable 1982), α‐acetylated tubulin clone 6‐11B‐1 (Sigma T6793) prepared from acetylated tubulin of *Strongylocentrotus purpuratus* was used at a 1:2000 dilution (Piperno & Fuller 1985), α‐β‐tubulin clone 2‐28‐33 (Sigma T5293) prepared from *S. purpuratus* sperm axonemes was used at a 1:2000 dilution (Banerjee et al. 1990) and antibody RN1 prepared using homogenates of *H. glaberrima* radial nerves as immunogen (Díaz‐Balzac et al. 2007) was used at a 1:50,000−1:100,000 dilution. In addition various rabbit polyclonal antibodies were used. These included α‐Pax6 (Abcam ab5790 Lot 464388, Cambridge, MA) prepared against the synthetic peptide C‐REEKLRNQRRQASNTPSHI corresponding to amino acids 267−285 of mouse PAX6 at a 1:100 dilution, α‐GFSKLYFamide No. 23 2i2s (second injection and second bleeding) (Díaz‐Miranda et al. 1995) prepared against a GFSKLYa synthetic peptide at a 1:1000 dilution, α‐phospho‐histone H3 (Upstate Biotechnology #06‐570, Lake Placid, NY) diluted at 1:250, α‐galanin 2i3s (second injection, third bleeding) diluted 1:1000 (Díaz‐Miranda et al. 1996), and α‐calbindin (Abcam ab11426 Lot 378854) prepared against 28 kDa calbindin‐D protein purified from rat kidney at a 1:500 dilution. While the use of most of these markers for labeling nervous components has been largely documented, two markers in particular (α‐Pax6 and α‐PH3) present some specific issues as to their specificity. We have shown in a recent publication (Díaz‐Balzac et al. 2014) that the labeling is indeed neuronal although the epitope that is recognized by these antibodies might be found in a molecule that might not be either Pax6 or PH3.

The secondary antibodies used were goat anti‐mouse GAM FITC (#AM10408, Biosource, Camarillo, CA) diluted 1:25; goat anti‐rabbit GAR FITC (#AR13408, Biosource) diluted 1:25; goat anti‐mouse GAM Cy3 (#115‐165‐068, Jackson ImmunoResearch Laboratories Inc., West Grove, PA) diluted 1:1000; and goat anti‐rabbit GAR Cy3 (#111‐165‐144, Jackson ImmunoResearch Laboratories Inc.) diluted 1:1000.

Phalloidin TRITC (Sigma Cat. #P1951) was also used in double labeling experiments to label the intestine muscle layers. Powder was diluted in methanol or dimethylsulfoxide at 5 mg/mL to prepare stocks. Dilutions of 1/50 were made to mix in equal amounts with primary antibody for a final concentration of 1/100.

### Nerve fiber quantification

**Figure 11:**
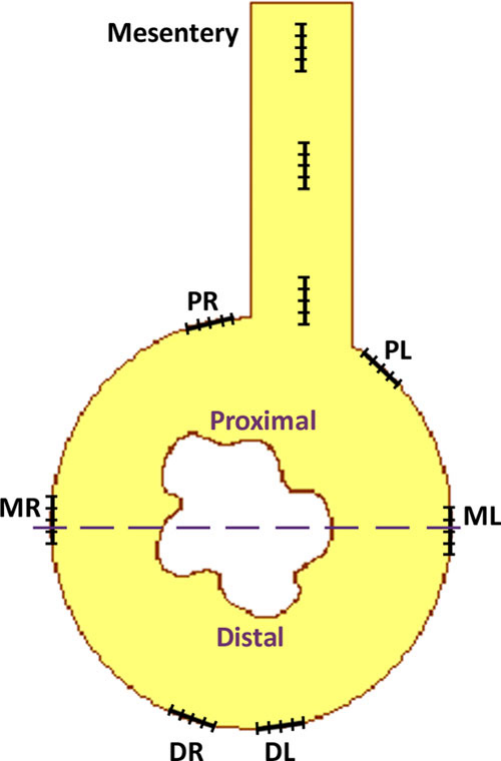
Quantification of nerve fibers in the intestinal regenerating rudiment. The mesothelia of normal or regenerating intestine were divided into areas proximal, medial, and distal relative to the attachment to the mesentery (proximal right [PR], proximal left [PL], medial right [MR], medial left [ML], distal right [DR], distal left [DL]). The microscope ruler scale (containing five sections) was placed over the region to be measured. Fiber presence was quantified by the number of sections in which fibers were found. Thus the range of measurements was from 0 to 5. A similar strategy was used to quantify fibers in the connective tissue, except that the regenerate was divided into two by an imaginary (dashed) line through the middle. The total number of fibers was counted in each half.

To quantify nerve fibers in the mesothelial layer, a strategy was developed in which the normal or regenerating intestine was divided into areas proximal, medial, and distal relative to the mesentery. Quantification was done using a Leitz Laborlux microscope with a 50× objective. The 100 μm microscope ruler scale was divided into five 20 μm sections and placed over the region to be measured. The quantification was based on the number of sections in which fibers were found. The range of measurements was from 0 to 5. The areas where the ruler was placed for quantification are shown in Figure 11. To determine differences in innervation, a ratio between the proximal and distal portions was calculated. A value close to 1 indicates no difference between the fiber distributions in the proximal versus distal regions of the regenerate. Values higher than 1 indicate that the nerve fiber distribution is higher in the proximal versus the distal portion of the regenerate. Fibers in the mesentery were also measured at three locations. At least three animals were used for each measurement.

To quantify nerve fibers in the connective tissue we divided the intestine or regenerating organ into two halves, proximal (adjacent to the mesentery) and distal (toward the coelomic cavity) (see Fig. 1). Three animals at each of the regenerating stages (7, 14, 21, 28 and 35 dpe) and three normal animals were used. The sections were labeled using the antibody against acetylated α‐tubulin, and all the nerve fibers in both halves were counted to calculate a proximal/distal ratio to determine if fibers were homogeneously distributed in the regenerating organ.

The appearance of nerve fibers that project from the mesothelium into the connective tissue during regeneration was also quantified. The strategy was similar to that used to quantify nerve fibers in the mesothelium. The microscope ruler was located over the boundary between the circular muscle layer and the dense connective tissue, and the numbers of nerve fibers that run perpendicular into the dense connective tissue within the ruler section were counted. Proximal, medial and distal sections were measured in three animals at each of the regeneration stages (7, 14, 21 and 28 dpe) and in three normal animals.
